# Otogenic Central Skull Base Osteomyelitis With Retropharyngeal Extension: A Case Report

**DOI:** 10.7759/cureus.22991

**Published:** 2022-03-09

**Authors:** Regina Jacobo-Pinelli, Jose Alberto Guerrero-Paz, Juan Antonio Lugo-Machado, Jose Alberto Arvizu-Flores, Karen Paulina Guerrero-Paz

**Affiliations:** 1 Otolaryngology, Hospital de Especialidades No. 2 Instituto Mexicano del Seguro Social (IMSS), Ciudad Obregón, MEX; 2 Neurological Surgery, Hospital de Especialidades No. 2 Instituto Mexicano del Seguro Social (IMSS), Ciudad Obregón, MEX

**Keywords:** esbl-positive e. coli, osteomyelitis, skull base osteomyelitis, retropharyngeal abscess, malignant otitis externa

## Abstract

Skull base osteomyelitis (SBO) is a life-threatening condition in patients with underlying comorbidities. Ear infections may spread through normal skull base fissures in this group of patients. However, its diagnosis is frequently delayed due to the unspecific clinical findings at onset, such as headache, with diverse cranial neuropathies later as the disease progresses. We present the case of a patient with otogenic skull base osteomyelitis complicated with retropharyngeal extension, treated with surgical drainage and broad-spectrum antibiotics directed toward extended-spectrum beta-lactamase (ESBL)-producing *Escherichia coli*, with recurrence of the infection three months later. With this case study, we aim to stress the importance of antimicrobial resistance and how it can preclude an otherwise favorable prognosis.

## Introduction

Skull base osteomyelitis (SBO) is a rare and life-threatening condition comprising a diagnostic and therapeutic challenge. This condition classically presents in the elderly and diabetic patients with nonspecific symptoms such as headache and cranial nerve palsies, mostly affecting the seventh cranial nerve (CN). Various diseases can mimic SBO, therefore requiring a high index of clinical suspicion [1,2]. Herein, we present the case of a patient with SBO secondary to an otogenic infection complicated with retropharyngeal extension.

## Case presentation

A 69-year-old female with a past medical history of type 2 diabetes, hypertension, and penicillin allergy was referred to our tertiary care center for the management of left chronic otitis media complicated with external otitis and ipsilateral peripheral facial nerve palsy ongoing for the past six months prior to our consult. The infection had been previously treated in the primary care setting with trimethoprim/sulfamethoxazole, erythromycin, and metronidazole. A computed tomography (CT) of the head was performed to rule out necrotizing otitis externa. Our initial management consisted of ciprofloxacin, corticosteroid, and a left ventilation tube insertion, with improvement of symptoms. At our three-month clinic follow-up, she reported progressive intermittent retro-ophthalmic pain with radiation to the occipital region, otalgia, tinnitus, and relapse of the purulent otorrhea.

Physical examination revealed a House-Brackmann III left peripheral facial nerve palsy. Otoscopy findings were notable for circumferential edema of the external third of the external auditory canal (EAC), purulent otorrhea, and granulation tissue at the level of the isthmus junction of the EAC; the ventilation tube was still present in the left tympanic membrane. Nasal endoscopy revealed a mass in the nasopharynx medial to the left Eustachian tube. Cranial nerve examination was unremarkable. An initial culture of the otorrhea was sent on admission to the hospital.

She was admitted for diagnostic workup and antibiotic therapy with levofloxacin and clindamycin, which was escalated to meropenem and vancomycin by the infectious disease specialist after antimicrobial susceptibilities resulted with ciprofloxacin-resistant and extended-spectrum beta-lactamase (ESBL)-producing *Escherichia coli* in the initial culture. Further laboratory studies are shown in Table 1.

**Table 1:** Laboratory studies at admission and during follow-up. HIV: human immunodeficiency virus

Laboratory studies	Admission	Third week
Hemoglobin (g/dL)	11.1	11.8
Leucocytes (10^3^/µL)	9	7.30
Neutrophil (10^3^/µL)	6.2	3.8
Lymphocytes (10^3^/µL)	1.7	2.3
Eosinophils (10^3^/µL)	0.4	0.3
Platelets (10^3^/µL)	361	262
Glucose (mg/dL)	109	154
Creatinine (mg/dL)	1	0.81
C-reactive protein (mg/dL)	4.24	4.05
Erythrocyte sedimentation rate (mm/hour)	36	13
Procalcitonin (ng/mL)	0.60	
Glycated hemoglobin A1C (%)	8.47	
HIV 1 and 2 antibodies	Negative	
Hepatitis B S antigen	Negative	

**Figure 1:**
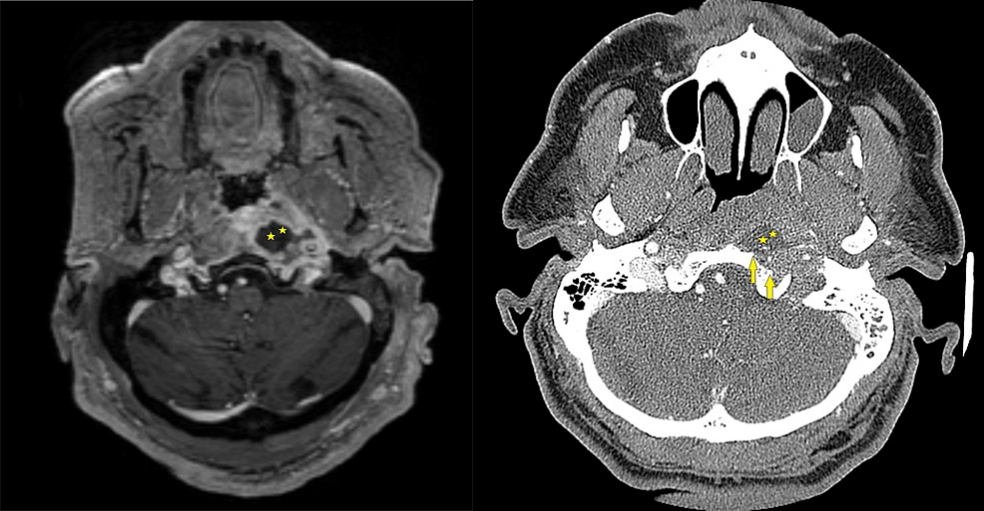
Axial MRI (left) and CT scan (right): anterior clival cortical bone loss (yellow arrows) and preclival rim-enhancing lesion (asterisks).

A contrast-enhanced CT scan of the head revealed findings of otitis media, clival demineralization, and a hypodense mass in the preclival region. Complementary magnetic resonance imaging (MRI) revealed a T1 hypointense and T2 hyperintense with peripheral rim enhancement lesion and diffusion restriction in diffusion-weighted imaging (DWI) (Figure 1).

**Figure 2:**
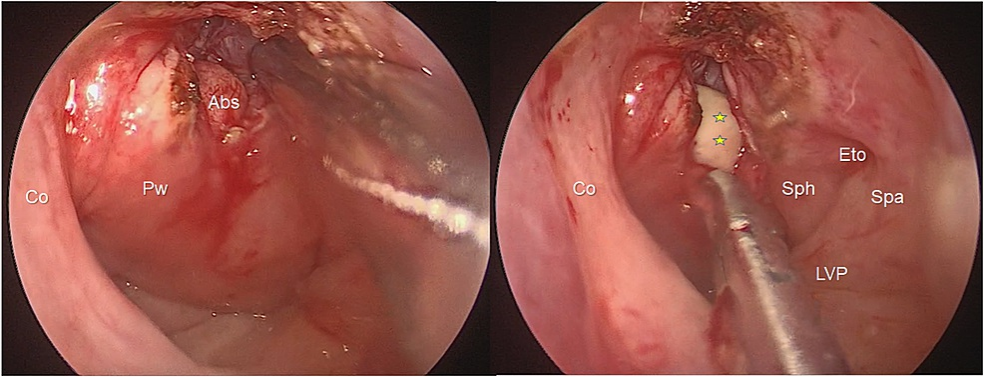
Surgical view of the nasopharynx: posterior wall approach for culture and biopsy. Abscess draining anterior to prevertebral muscles (asterisks). Co: choanal arc, Pw: posterior wall of the nasopharynx, Abs: abscess, Sph: salpingopharyngeal fold, Spa: salpingopalatine fold, LVP: levator veli palatini, Eto: Eustachian tube orifice

An endoscopic approach to the nasopharynx and the left EAC was used for drainage and to obtain biopsy specimens and further tissue cultures (Figure 2). An unspecific ulcered chronic inflammatory response without dysplasia, malignancy, or granulomatous findings was revealed; the cultures were negative for bacterial, fungal, or mycobacterial organisms. After endoscopic drainage, treatment with meropenem/vancomycin was continued for three subsequent weeks in a joint decision with our infectious disease specialist before discharge. Single-photon emission computed tomography (SPECT)/CT scan with technetium-99m two weeks after the initiation of treatment was negative for osteoclastic activity. The patient experienced resolution of otorrhea and headaches with antibiotic therapy and drainage of the nasopharyngeal mass. At her six-month follow-up appointment, the patient reported the same symptoms, and we noted clinical relapse with findings of purulent retronasal discharge, headache, and left vocal cord palsy; however, the patient declined further therapeutic management.

## Discussion

SBO is a rare entity characteristic of immunocompromised patients with nonspecific symptoms in relation to the affected structures [3]. It is classified as typical (otogenic, traumatic, or postsurgical) and atypical (also known as central due to clivus involvement) [4]. In immunocompromised patients, the infection usually extends inferiorly from the osteocartilaginous junction of the external auditory canal to the vertical fissures of Santorini, to the Haversian system, affecting the lower nerve foramina. The facial nerve is usually the first to be involved at the exit of the stylomastoid foramen. The infection can also spread medially to the jugular foramen (with involvement of cranial nerves IX, X, and XI) and petrous apex (with involvement of cranial nerves V and VI); anteriorly to the parotid, masticator, or parapharyngeal spaces; or even intracranially through the petroclival synchondrosis [3].

Among the causative microorganisms, *Pseudomonas aeruginosa* is the most common infectious agent; nevertheless, other Gram-positive bacteria such as *Staphylococcus*, *Streptococcus*, or even fungi have been described as etiological agents [1,5]. *Escherichia coli* as species produce extraintestinal disease in immunocompromised hosts; this infectious agent can be resistant to ciprofloxacin [6]. Acquired immunocompromise state due to diabetes mellitus has been associated with multiple factors: denervation, reduced perfusion, and impaired bactericidal function. Aging has also been associated with a reduction in T cell response [7]. Albeit rare, our patient had an infection with an opportunistic organism paired with a reduction of immunity, both advanced age and uncontrolled diabetes contributing to the severity of the infection. We did not find a previous case of otogenic skull base osteomyelitis due to extended-spectrum resistant *Escherichia coli* reported in our literature review.

Early imaging is crucial, as the unspecific clinical findings of this entity can delay diagnosis, and further complications can easily appear [8]. The diagnostic armamentarium includes a non-enhanced CT scan that showcases late demineralization findings, contrast-enhanced CT that permits identification of fat space disruption or tissular edema, magnetic resonance imaging with gadolinium that offers superior differentiation of soft tissue involvement [4,8], and nuclear imaging with technetium-99m or gallium-67, the former identifying osteoblastic activity and the latter being more specific to infection. These studies have also been described in monitoring the outpatient setting [4].

Broad-spectrum antibiotic therapy with antipseudomonal coverage is considered the mainstay of treatment. Current evidence recommends a longer duration of intravenous antibiotics, although this is still somewhat controversial. Regarding surgical management, evidence is still inconclusive, but evidence suggests that early debridement of necrotic tissue improves clinical outcomes and reduces inflammation burden. We opted for a four-week intravenous antibiotic therapy course and outpatient treatment for two more weeks with ciprofloxacin [2,9].

Our patient presented with recurrent episodes of headache and persisting otorrhea, which prompted further diagnostic workup. Cultures and contrast-enhanced imaging were crucial to the decision-making in this case. There was an absence of bone destruction on the external, middle, or internal ear, which made it more difficult to reach a diagnosis until bone erosion was evident in the clival region. With this case study, we aim to highlight the importance of maintaining a high clinical index of suspicion, obtaining cultures to guide antimicrobial therapy, and prompt imaging studies to guide duration, treatment, and response during the follow-up period.

## Conclusions

Skull base osteomyelitis is a poorly described clinical entity in our environment. It requires a high clinical suspicion to be diagnosed promptly, reducing morbidity and mortality from complications related to this condition. An accurate diagnosis in a timely fashion is imperative to improve prognosis in patients with SBO. With this report, we aim to contribute to filling in the gaps in knowledge in the decision-making processes of these challenging cases with the goal of improving outcomes in SBO and avoiding recurrence and complications.
